# Fifteen Years of Emergency Visits for Whiplash Injuries: Impact of COVID-19 and Campaign to Reduce Minor Injury Admission

**DOI:** 10.3390/life15070987

**Published:** 2025-06-20

**Authors:** Harpa Ragnarsdóttir, Kristín Rut Arnardóttir, Kristín Briem, Micah Nicholls, Hjalti Már Björnsson

**Affiliations:** 1 Department of Physical Therapy, Faculty of Medicine, School of Health Sciences, University of Iceland, 102 Reykjavík, Iceland; kra31@hi.is (K.R.A.); kbriem@hi.is (K.B.); 2 Elja Physical Therapy, 220 Hafnarfjörður, Iceland; 3 Össur ehf, 110 Reykjavík, Iceland; mnicholls@ossur.com; 4 Faculty of Medicine, University of Iceland, 101 Reykjavík, Iceland; hjaltimb@hi.is; 5 Emergency Department, Landspitali—The National University Hospital of Iceland, 101 Reykjavík, Iceland

**Keywords:** neck pain, cervical spine, motor vehicle accidents, car collision, car accidents, whiplash-associated disorders, COVID-19

## Abstract

Whiplash-associated disorder (WAD) is common following motor vehicle collisions (MVCs). The yearly incidence rate in the Western world has been reported to be around 300 per 100,000 habitants, but no publications have examined yearly incidence across a period that includes the COVID-19 pandemic. A retrospective, epidemiological study was conducted in Iceland involving data from the University Hospital and the healthcare centers for the Capital Region for all individuals who visited the emergency department during 2010–2024 due to TAs, with a diagnosis indicating whiplash injury. The yearly incidence rate was calculated and presented per 100,000 person-years and analyzed by age, sex, months, and weekdays. The overall incidence of whiplash injuries was 267 per 100,000 person-years, greater for females than males (*p* < 0.001) with a significant effect of age (*p* < 0.001), the greatest rate being seen in young adulthood. A significant effect of time was seen across the study period (*p* < 0.001) due to a sharp decline between 2016 and 2020, followed by a continued low yearly incidence rate, with the smallest one seen in 2024 (78 per 100,000). Despite an increase in MVCs worldwide, the incidence of whiplash injuries following MVCs has declined significantly over the past decade. This trend may reflect shifts in injury patterns, healthcare-seeking behavior, or reporting practices.

## 1. Introduction

Neck pain is a prevalent condition causing significant pain, disability, and economic burden, affecting 203 million people in 2020 and projected to impact 269 million by 2050 [1]. The greatest age-adjusted prevalence in 2017 was observed in Western Europe, East Asia, North Africa, and the Middle East, with the prevalence in Norway, Finland, and Denmark ranking the highest among individual countries [1]. Whiplash, resulting from the sudden acceleration–deceleration of the head and neck, can cause various injuries to the cervical spine, including bones, facet joints, ligaments, disks, and vertebral arteries [2,3]. While many individuals recover from a whiplash injury within weeks, some go on to develop persistent symptoms commonly referred to as whiplash-associated disorders (WADs) [4], a cluster of biopsychosocial symptoms [5,6,7], that tend to become chronic [2].

The yearly incidence of whiplash injuries following motor vehicle collisions (MVCs) in Western Europe and North America is estimated at around 300 per 100,000 inhabitants [8,9,10]. Since 1980, emergency department (ED) visits due to MVCs have increased in the Western world [10], with an annual socioeconomic cost of approximately EUR ten billion in Europe alone [10,11,12,13]. Studies from Sweden and the U.S. report incidence rates of 235 and 328 per 100,000 person-years, respectively, with females accounting for a greater proportion of incidence rates (59% and 52%, respectively) [14,15] and a peak in the incidence rate observed among individuals aged 20–24 (>450 per 100,000 person-years) [14]. While incidence remained stable in the early 2000s [9,11], to the authors’ knowledge, there are no recent studies investigating the effects of continued efforts to improve safety on the incidence of whiplash after MVCs.

Emergency department strain and wait times have increased over the past two decades, contributing to growing overcrowding, which is increasingly recognized as a global public health concern [16,17,18]. Overcrowding results from the interplay of input (e.g., increased patient arrivals), throughput (e.g., delays within the ED), and output (e.g., boarding of admitted patients due to lack of inpatient beds) [17,18]. Increasing after-hours access to primary care [19,20,21] may help to reduce the likelihood of ED utilization [18]. Promoting such a shift in care was attempted in Iceland in 2018 using a social media campaign advising individuals with minor injuries to use same-day scheduling at their local healthcare centers instead of going to the ED. Gathering restrictions during the COVID-19 pandemic are also likely to have influenced ED utilization, in part due to less traffic (a greater proportion of people working from home). Research on the COVID-19 pandemic’s influence on traffic has suggested an increase in serious collisions [22,23] but a decrease in crashes and injuries overall [23]. Updated information on the epidemiology of whiplash injuries due to MVCs is called for, considering recent changes in patient care and the potential impact of the pandemic. The results may provide valuable insights for other healthcare systems and may be utilized to raise awareness about whiplash injuries and WADs. Although not all individuals who experience a whiplash injury go on to develop WADs, understanding the population-level incidence of whiplash may aid in identifying potential patterns and risk factors associated with chronic symptom development. Such data are valuable for informing early intervention strategies and guiding healthcare planning related to trauma recovery and long-term outcomes.

The purpose of this study was to (1) investigate the number of individuals diagnosed with a whiplash injury after an MVC in the Capital Region in Iceland between 2010 and 2024; (2) assess the impact of a campaign in 2018 by the University Hospital and the healthcare centers of the Capital Region (HCCs) intended to redirect individuals with minor injuries away from the ED to alleviate overcrowding; and (3) assess the impact of COVID-19 restrictions on ED visits. We hypothesized that sex- and age-dependent differences in incidence rates would be found, with greater numbers for females and younger age groups. Additionally, it was hypothesized that the campaign would result in a decrease in ED visits for whiplash injuries and a concurrent increase in visits to HCCs and that the incidence rate would be temporarily reduced during the timeframe of COVID-19 restrictions.

## 2. Materials and Methods

Data from January 2010 to December 2024 were obtained from the ED at the University Hospital, which serves the greater Capital Region in Iceland, containing 64% of the nation’s inhabitants [24]. Data from all HCCs in the Capital Region (*N* = 19) were obtained from 2010 to 2023. All individuals registered on arrival with an International Classification of Primary Care code (ICPC) of “traffic accident” and an International Classification of Disease, 10th revision (IDC-10), S13.4 (sprain and strain of cervical spine) or S13.6 (sprain and strain of joints and ligaments of other and unspecified parts of neck) [25] were, in this study, considered to have sustained a whiplash injury and included for analysis. In addition to age and sex, the date, time of day, weekday, month, and year of admission were recorded. The same data were obtained from the HCCs of the capital area, although the date of injury was not available, which precluded the determination of whether injuries were acute vs. chronic.

To determine injury incidence, the total population in each year for the region was obtained for the same timeframe. The region was defined as the City of Reykjavík and the six smaller municipalities considered part of the so-called “greater Capital Region” [26]. Additionally, to put the results into context, data were obtained from the ED detailing all ED visits during the study period, categorized into triage categories 0–5, with whiplash injuries most likely falling under category 4 (moderate injuries). The Icelandic Transport Authority provided the number of registered MVCs (with and without documented injuries) in the Capital Region for the study period. This study was approved by the National Bioethics Committee (VSNb202103001 l/03.0I) and by the Ethics Committees of the University Hospital and the HCCs.

Data from the ED and the HCCs were analyzed separately due to the possibility that the HCC dataset may have contained both acute and chronic cases. The yearly incidence of whiplash injuries as per ICD-10 S13.4 and S13.6 was presented per 100,000 person-years by using population data from the capital area and was assumed to fit a Poisson distribution. Data from the HCCs were evaluated specifically to detect any changes in the number of visits to the HCCs coinciding with changes observed in the ED data and to monitor changes during COVID-19 when individuals were encouraged not to visit the ED unless in extreme cases. A generalized additive model (GAM) was used to model the relationship between age, sex, and whiplash injuries per age across the time period. For the GAM, incidence rates were assumed to fit a Poisson distribution, and where significant interactions occurred between age and sex, separate models were created for each sex. The Wilcoxon test was selected as a non-parametric alternative to the *t*-test due to a skewed distribution of age data between sexes. The difference between the sexes in overall whiplash incidence was tested using the Poisson test (R package stats package version 3.6.2). No adjustments were made for multiplicity. The distribution of the average number of whiplash injuries for each month and weekday across the study period was also assessed. Category 4 visits were calculated as a proportion of all ED visits across the period, as were the number of reported MVCs. All data analyses were performed using R statistical software (versions 4.4.1) and Excel spreadsheets.

## 3. Results

**Figure 1:**
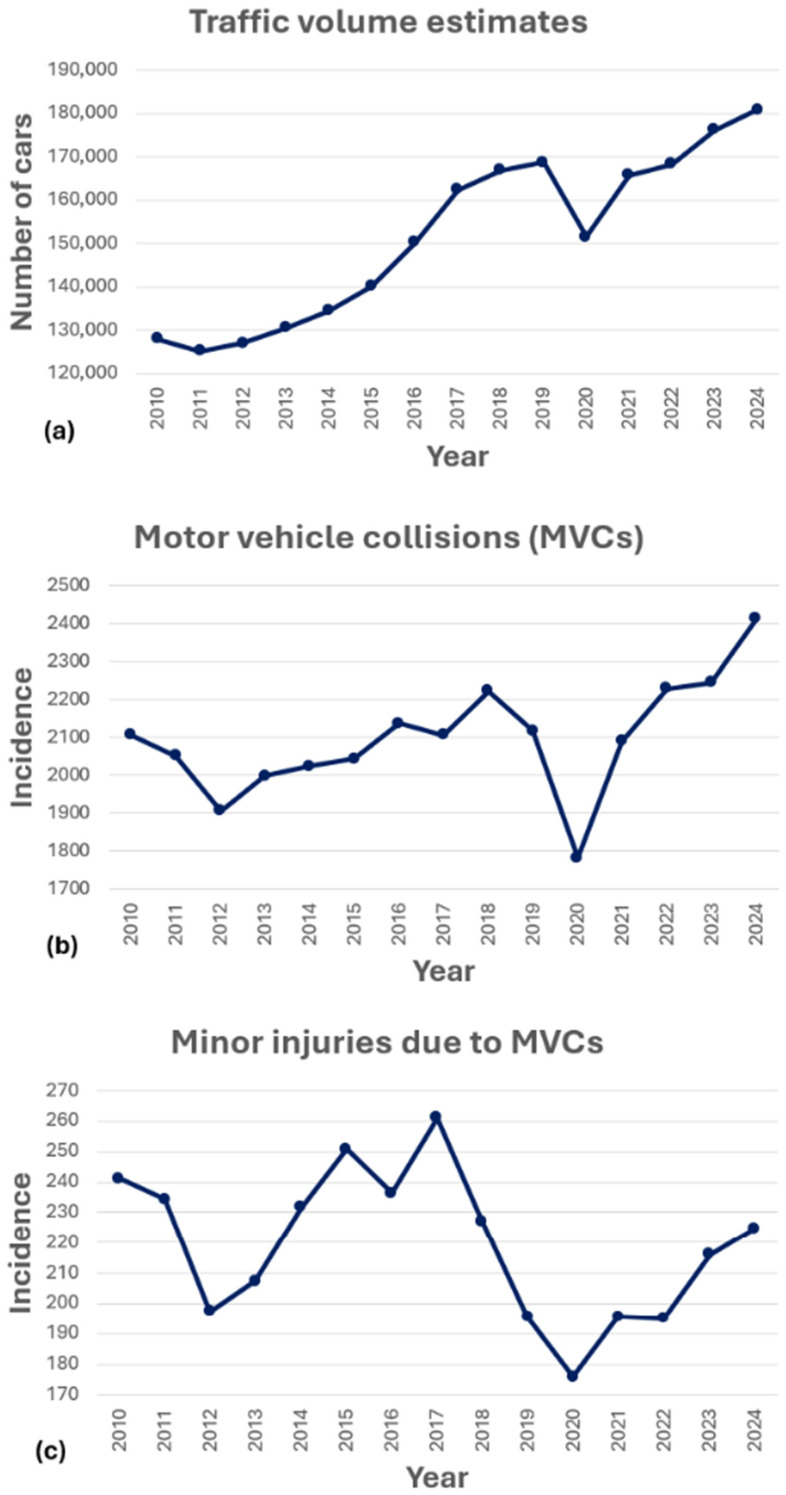
(**a**) Traffic volume trends based on three primary traffic counters, (**b**) yearly motor vehicle collision (MVC) incidence per 100,000 person-years, and (**c**) yearly minor injury incidence due to MVCs per 100,000 person-years, in Capital Region, 2010–2024.

Figure 1 illustrates the yearly (a) traffic volume estimates, (b) MVC incidence as registered by the Icelandic Transportation Authority, and (c) minor injury incidence following MVCs registered by the Icelandic Transportation Authority in the Capital Region during the study period. Between 2010 and 2024, a total of 68,763 MVCs were registered, resulting in 8368 documented injuries. Of these injuries, 7146 were classified as minor, 41 were fatalities, and the remaining 1181 were classified as major injuries. The classification of injuries followed the international framework established by the United Nations Economic Commission for Europe, Geneva. According to this classification, major injuries refer to those that require medical treatment and cause an impairment of mobility or daily function (e.g., fractures, deep wounds), while minor injuries involve superficial harm (e.g., bruises, abrasions) with limited or no functional impact [27].

The incidence of MVCs and the total average traffic per year show an overall upward trend throughout the study period. During the same time period, a total of 21,608 individuals came to the ED following TAs, with a total of 8256 (38%) diagnosed with whiplash injuries (S13.4 and S13.6). A steady decline over time was seen in the proportion of individuals receiving whiplash diagnosis at the ED (from 45% in 2010 to 23% in 2024; Table 1). Those injured with whiplash injury were on average 33.9 years old and 58% female (range 53–62% throughout the years). The difference in the number of whiplash cases before and after reaching the legal driving age (17 y.o.) was substantial. The total number of whiplash cases in 16–17-year-olds was 30, while that for 18–19-year-olds was 768 (Table A1, Appendix B).

**Figure 2:**
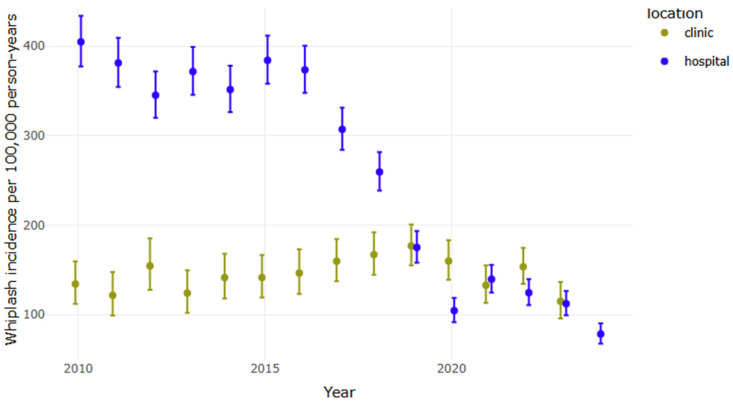
Overall yearly incidence by location of diagnosis (emergency department (hospital) vs. healthcare centers (clinic)).

The vast majority of whiplash cases were seen at the ED, with only a small portion seen at the HCCs (Figure 2). The incidence of whiplash injury, per 100,000 person-years over the study period, was 253, which was substantially greater for females compared to males (293 vs. 212 per 100.000 person-years, respectively, *p* < 0.001). Cases per 100,00 person-years ranged from 345 to 405 from 2010 to 2016, after which there was a marked drop (Figure 2), with the smallest number registered in 2024 (78 cases per 100,000). The greatest year-to-year relative decreases in incidence were observed between 2019 and 2020 (−40.3%), 2018 and 2019 (−32.6%), and 2023 and 2024 (−30.2%); year-to-year differences between other years ranged from −17.8% (between 2016 and 2017) to +34% (between 2020 and 2021).

**Figure 3:**
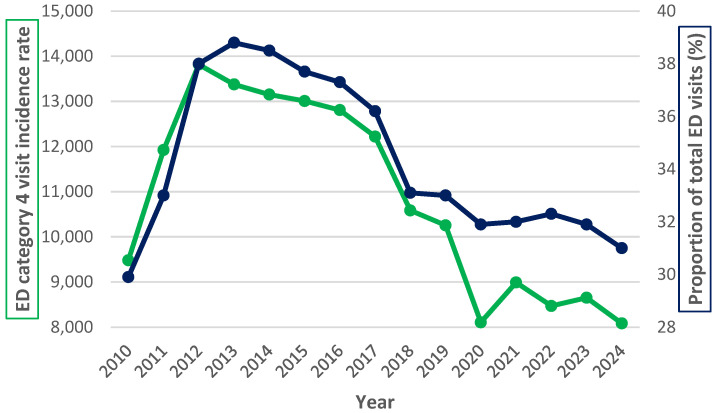
Yearly incidence rate of emergency department (ED) category 4 visits per 100,000 person-years in Capital Region (blue line) and their percentage of all ED visits (green line), 2010–2024.

Yearly ED visits in category 4 (moderate severity, including whiplash injuries) accounted for 30–39% of all ED visits over the study period (Figure 3). Although the incidence rates of category 4 visits declined steadily after 2014, the proportion of total ED visits remained relatively constant.

**Figure 4:**
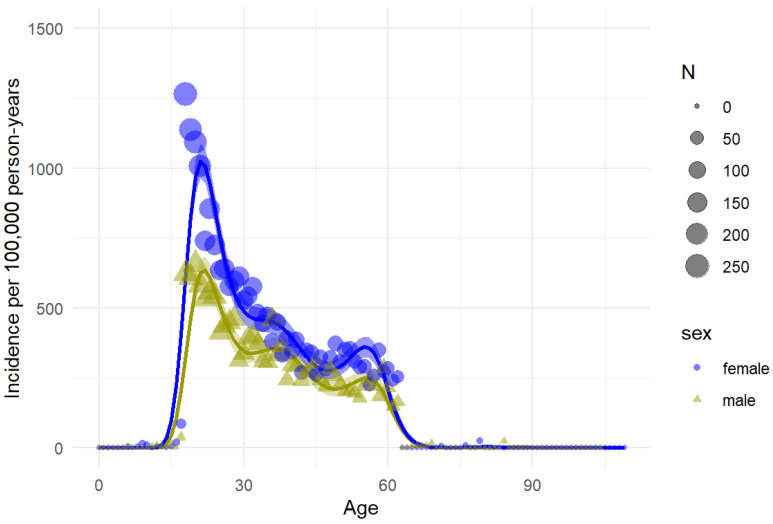
Distribution of whiplash injuries by sex and age from 2010 to 2024. Incidence rates were calculated as number of new cases per 100,000 inhabitants per year, based on age- and sex-specific population counts in Capital Region. Shaded areas represent 95% confidence intervals.

An analysis of the 2010–2024 average incidence of whiplash injuries across all ages for males and females showed an overall main effect of age (*p* < 0.001), due to the large peak during their late teens and twenties (Figure 4), and a significant interaction for sex and age (*p* < 0.001; Figure 4).

**Figure 5:**
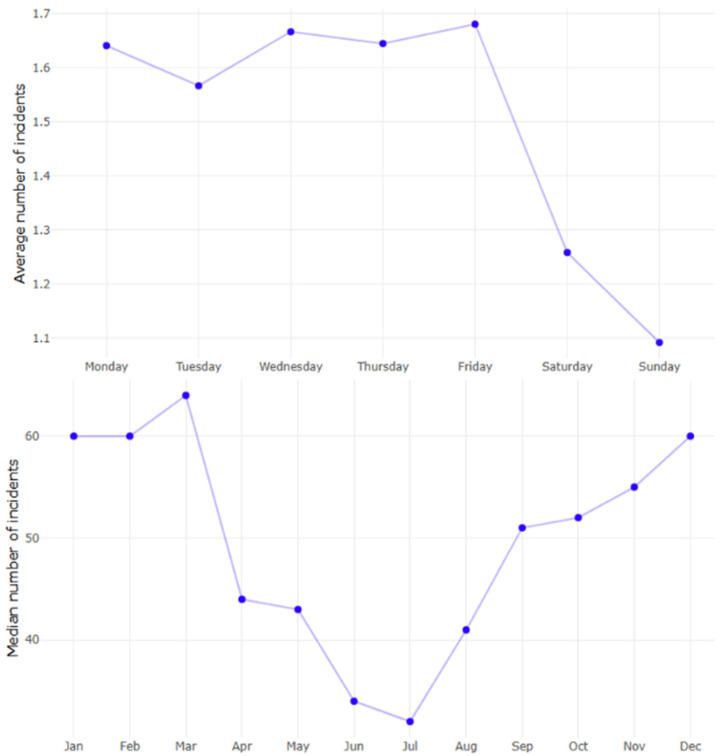
The average number of individuals who had sustained whiplash following motor vehicle collisions by weekdays and the median number by months from 2010 to 2024.

The average number of injuries varied significantly throughout the week (*p* < 0.001; Figure 5) and between weekdays versus weekends (*p* < 0.001), revealing the smallest average number of incidents on Sundays (mean 1.09, SD: 1.40) and the largest on Fridays (mean 1.68, SD: 1.78). Additionally, the average number of injuries varied significantly throughout the year (*p* = 0.008). The winter months, December through March, had the greatest average number of whiplash injuries (median 60–64 incidents per month), while the smallest were in June and July (median 34 and 32 incidents per month, respectively, Figure 5).

## 4. Discussion

Although whiplash injuries are common and costly, longitudinal data on incidence trends is lacking in recent years, and research on the impact of public health campaigns and global disruptions such as the COVID-19 pandemic is scarce. The gap in the literature hinders effective health planning and resource allocation. The current study aimed to address this gap by analyzing a 15-year dataset of emergency department visits for whiplash injuries in Iceland’s Capital Region, covering a period that includes the implementation of a healthcare redirection campaign and the COVID-19 pandemic. The primary aim was to examine temporal trends in whiplash incidence following MVCs and assess the potential impact of external events on healthcare utilization. Given increasing global traffic volumes [28] and ongoing pressures on emergency services [16,17,18], such insight is essential to guide policy and improve care delivery. The most important results of this study showed that, despite a steady increase in traffic and the incidence of MVCs in the Capital Region, the proportion of passengers with minor injuries relative to the number of MVCs remained mostly unchanged. Meanwhile, a significant reduction in the number of individuals diagnosed with cervical sprain/strain (ICD-10 S13.4/S13.6), used in this study as a proxy for whiplash injury, was seen over the study period. Notably, the 72% reduction in the incidence of documented whiplash injuries between 2016 and 2020 was much greater than the 36.7% decrease in category 4 ED visits for moderate injuries during the same period.

The greatest whiplash incidence rate of hospital-diagnosed whiplash was observed in 2010, with 405 cases per 100,000 person-years. Although incidence rates fluctuated in the years that followed, a consistent downward trend began after 2016, even though minor injuries reported with registered MVCs did not start to decrease until after 2017. This decline preceded both the COVID-19 pandemic and the launch of the 2018 healthcare campaign intended to redirect patients with minor injuries away from the ED. However, not surprisingly, the sharpest year-to-year reduction (40.3%) did coincide with the onset of the COVID-19 pandemic in Iceland in 2020. Additionally, the particularly sharp 32.6% reduction noted between 2018 and 2019 suggests that the 2018 redirection campaign may have contributed to changes in healthcare-seeking behavior. The observed reduction in ED visits for whiplash injuries may in part reflect the influence of the 2018 healthcare redirection campaign, although causal relationships cannot be established based on registry data alone. Notably, this decline in ED visits was not accompanied by an increase in visits to HCCs during the same period, which raises the possibility that some individuals may have sought care outside the settings covered in this study or not at all, potentially reflecting changes in healthcare-seeking behavior or accessibility. However, some may have instead sought treatment at an independently operated after-hours urgent care service, for which data were unavailable in this study. These findings highlight the potential impact of targeted healthcare initiatives on patient distribution and resource allocation in emergency services. This is particularly noteworthy given that traffic volume in the capital area increased by 41% over the study period, according to the Icelandic Transport Authority [29], probably contributing to the increase in MVCs during the study period. Interestingly, while the number of MVCs and associated minor injuries began to rise again after 2020, the number of hospital-diagnosed whiplash cases remained relatively small, further suggesting potential changes in healthcare-seeking behavior, injury patterns, or reporting practices.

In addition to the 2018 campaign, other factors may have contributed to the observed reduced whiplash incidence. One possibility is advancements in vehicle design and safety features, which may have reduced the likelihood of sustaining a neck injury in a collision. Whiplash-reducing head restraint systems have been shown to result in a statistically significant reduction in whiplash injuries sustained by drivers and front-seat passengers following a rear-end collision (−11.6%) [30]. Another potential explanation is a change in diagnostic coding practices, as coding was moved from medical secretaries to medical doctors in 2012–2014. However, the effects from these changes should have been apparent before 2016.

The greater proportion of females among whiplash injury cases is consistent with previous research [14,15]. Females seem to be more prone to whiplash injuries compared to males, despite the fact that young-adult males show an increased risk of crash compared to females [31]. Difference in neck muscle strength, anthropometry, and biomechanical responses may explain these gender differences [31,32,33]. Additionally, despite known anatomical differences, automobile safety features have been predominantly based on male crash test dummies and biased towards males when assessing inequities, potentially increasing the injury risk for female drivers and passengers [34,35].

The pattern of injury incidence across age is mostly in line with previous studies [9,14,15,36]. Notably, the legal age to obtain a driver’s license in Iceland is 17 years old [37], which likely accounts for the increase in whiplash injuries for that age group of young, inexperienced drivers. Although our data did not discriminate between injured drivers versus injured passengers, the sharp increase in incidence for those older than 17 years old suggests that inexperience behind the wheel may be a contributing factor. Furthermore, the significant increase in injuries between the 16–17- and 18–19-year-olds underscores the role of driving exposure in whiplash risk. The small incidence rate for the youngest group may be explained in part by children’s safety regulations, such as rear-facing car seats for kids up to 3–4 years of age, with a subsequent switch to forward-facing car seats. In Europe, it is also mandatory that a child under 135 cm (about 10 years old) must be in approved safety equipment suitable for their height and weight [38,39,40]. Interestingly, our analysis revealed a significant sex-by-age interaction in the injury incidence of whiplash following MVCs. Females exhibited a sharper increase and a much greater incidence during young adulthood, after which the difference diminished. Whether this is due to gender- and age-dependent factors during maturation is worthy of further research.

**Figure A1:**
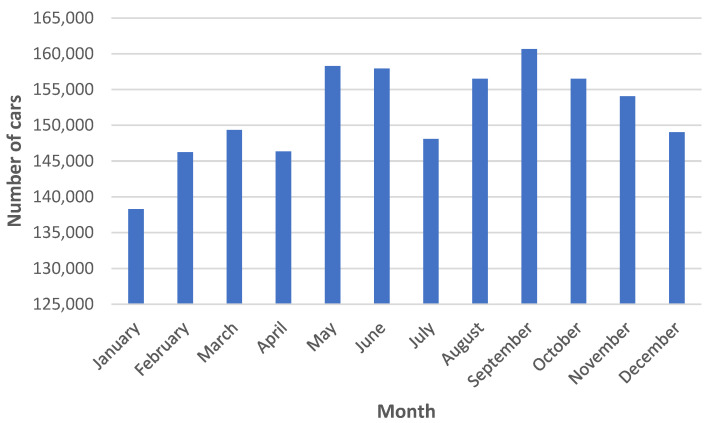
Total average traffic in the Capital Region, by month. Based on three primary traffic counters.

The differences seen between weekdays likely reflect the fact that fewer people attend work and school over the weekends. Monthly comparisons showed that the winter months, particularly December through March, had the greatest median number of injuries, whereas the smallest were recorded in June and July. Interestingly, these injury incidence peaks occurred during a period when available traffic volume data in the capital area [29] indicated relatively small traffic levels in January through April (Figure A1, Appendix A). This suggests that the increased risk of injury in winter months may be more closely associated with seasonal factors such as hazardous road conditions, ice, and limited daylight, rather than traffic exposure alone.

### Strengths and Limitations

The long study period of 15 years that includes and surpasses the COVID-19 pandemic allows for unusual analyses regarding the yearly incidence of whiplash pre- and post-COVID-19. This is the first study to describe changes in the incidence of whiplash injuries across a 15-year period that includes both the onset and aftermath of the COVID-19 pandemic. The data reflects the majority of a nation, as the greater Capital Region encompassed 63–64% of the population in Iceland during the study period [41]. Limitations that should be acknowledged include the possibility that some individuals with whiplash injuries may have been registered with an ICD-10 code within the S10-19 category (“injuries to neck”) [25] other than S13.4 and S13.6 and, conversely, that not all individuals registered with S13.4 or S13.6 necessarily sustained a whiplash injury. These codes, commonly used to indicate cervical sprain/strain, are diagnostically vague and do not specify the exact anatomical structures involved. Moreover, the clinical tests used to support such diagnoses have shown limited reliability and validity [42]. In addition, although individuals assigned these codes were classified as having sustained a whiplash injury, the codes do not reflect the full clinical spectrum of whiplash-associated disorders (WADs), and the findings should be interpreted with this limitation in mind. Some individuals may have visited both the ED and the HCCs; however, data from the HCCs were only used to assess whether HCC visits increased at the time of the decline in ED visits and thus did not contribute to incidence calculations. Additionally, data from the HCCs in Iceland did not distinguish between acute and chronic injuries, but the number of cases recorded at these centers was relatively small and remained fairly stable during the decline in visits to the ED. Despite repeated attempts, the researchers were unable to obtain data from “Læknavaktin”, an independently operated urgent care service in the capital area that is open in the evenings and on weekends, serving individuals with health concerns that cannot wait until the next working day. The absence of these data may have introduced bias, as individuals with whiplash injuries may have sought care at Læknavaktin rather than the ED or HCCs. While this study examines individuals registered with ICD-10 codes S13.4 and S13.6 following MVCs based on the total average population per year in the capital area, it is important to acknowledge that individuals residing outside the capital area, as well as tourists, also seek medical attention at the ED following MVCs. Finally, while injury severity following MVCs was classified according to the Economic Commission for Europe guidelines, they did rely on registry data and clinical judgment.

## 5. Conclusions

Despite increasing traffic volumes and more registered MVCs in the Capital Region over the past 15 years, a marked and disproportionate decline in hospital-diagnosed whiplash injuries was observed, exceeding the reduction seen in other moderate injury presentations. While a limited corresponding rise in visits to the HCCs was observed, the largest reductions coincided with the 2018 redirection campaign and the onset of COVID-19, suggesting that these external events may have influenced healthcare-seeking behavior. As hypothesized, females and younger individuals were more frequently diagnosed with whiplash, consistent with the prior literature. Although registry data limit causal interpretation, these findings highlight the value of long-term surveillance and suggest a need for further investigation. Investigations should be conducted both within Iceland to clarify whether the reduction reflects a true decrease in incidence, changes in diagnostic or reporting practices, or a shift in where individuals seek care and internationally to determine whether similar trends are evident elsewhere.

## Figures and Tables

**Table 1:** Number of all registered motor vehicle collisions (MVCs) in capital area, number of all registered emergency department (ED) visits due to MVCs, and number of all registered ED visits with ICD ^1^ S13.4 or S13.6 indicating whiplash injury.

Years	Total MVCs	Total MVCs with Injuries (% of Total MVCs)	All ED Visits with ICPC ^2^ “Traffic Accident”	Whiplash (% of Total MVCs)
2010	4193	428 (10)	1786	806 (19)
2011	4065	393 (10)	1746	755 (19)
2012	3811	359 (9)	1627	690 (18)
2013	4051	389 (10)	1755	753 (19)
2014	4160	419 (10)	1692	722 (17)
2015	4246	449 (11)	1920	798 (19)
2016	4503	450 (10)	1955	787 (17)
2017	4534	475 (10)	1745	661 (15)
2018	4900	413 (8)	1688	572 (12)
2019	4756	376 (8)	1243	393 (8)
2020	4063	368 (9)	812	238 (6)
2021	4841	442 (9)	965	323 (7)
2022	5267	475 (9)	786	294 (6)
2023	5425	489 (9)	1042	271 (5)
2024	NA ^3^	NA	846	193 (NA)

^1^ ICD: International Classification of Diseases; ^2^ ICPC: International Classification of Primary Care, ^3^ NA: Not Applicable.

## Data Availability

Data can be accessed from the lead author upon reasonable request.

## Appendix B

**Table A1:** The total number of whiplash incidents by age and sex in the Capital Region, 2010–2024.

AGE	Sex	No. of Injuries	Person-Years	Incidence ^1^
0	female	0	20,996.0	0
0	male	0	22,419.0	0
1	female	0	20,945.0	0
1	male	0	22,264.5	0
2	female	0	21,103.0	0
2	male	0	22,285.5	0
3	female	0	21,030.0	0
3	male	0	22,186.0	0
4	female	0	21,015.5	0
4	male	0	21,976.0	0
5	female	0	20,936.0	0
5	male	0	21,908.0	0
6	female	1	20,899.0	5
6	male	1	21,874.0	5
7	female	0	20,957.0	0
7	male	0	21,779.0	0
8	female	1	20,979.5	5
8	male	0	21,672.5	0
9	female	3	21,059.5	14
9	male	0	21,703.0	0
10	female	2	21,059.0	9
10	male	0	21,723.5	0
11	female	0	20,933.0	0
11	male	1	21,621.0	5
12	female	1	20,821.5	5
12	male	2	21,588.0	9
13	female	1	20,775.5	5
13	male	1	21,562.5	5
14	female	0	20,657.5	0
14	male	1	21,390.5	5
15	female	1	20,447.5	5
15	male	3	21,266.5	14
16	female	4	20,456.5	20
16	male	0	21,292.0	0
17	female	18	20,515.5	88
17	male	8	21,330.5	38
18	female	261	20,673.0	1263
18	male	134	21,472.5	624
19	female	240	21,101.0	1137
19	male	133	21,850.0	609
20	female	236	21,586.0	1093
20	male	147	22,392.5	656
21	female	222	22,071.5	1006
21	male	133	22,900.0	581
22	female	167	22,545.0	741
22	male	124	23,396.5	530
23	female	196	22,978.0	853
23	male	133	23,901.5	556
24	female	169	23,316.5	725
24	male	131	24,213.5	541
25	female	151	23,755.0	636
25	male	101	24,590.0	411
26	female	155	24,154.0	642
26	male	110	25,212.5	436
27	female	141	24,476.5	576
27	male	116	25,797.0	450
28	female	148	24,685.0	600
28	male	121	26,260.0	461
29	female	152	24,793.0	613
29	male	83	26,678.5	311
30	female	129	24,447.5	528
30	male	89	26,432.5	337
31	female	131	24,201.5	541
31	male	103	26,249.5	392
32	female	137	23,817.5	575
32	male	103	25,710.5	401
33	female	113	23,432.5	482
33	male	94	25,293.0	372
34	female	103	23,167.5	445
34	male	77	24,820.0	310
35	female	108	22,805.0	474
35	male	75	24,376.5	308
36	female	86	22,517.0	382
36	male	110	24,026.0	458
37	female	101	22,468.5	450
37	male	79	23,743.5	333
38	female	75	22,433.5	334
38	male	86	23,613.5	364
39	female	87	22,256.0	391
39	male	57	23,559.5	242
40	female	76	22,089.5	344
40	male	69	23,295.0	296
41	female	84	21,828.0	385
41	male	67	22,951.0	292
42	female	58	21,565.0	269
42	male	54	22,540.5	240
43	female	75	21,524.5	348
43	male	60	22,262.0	270
44	female	73	21,412.5	341
44	male	60	22,019.0	272
45	female	56	21,348.0	262
45	male	66	21,708.0	304
46	female	69	21,288.0	324
46	male	52	21,490.0	242
47	female	60	21,231.0	283
47	male	47	21,333.5	220
48	female	68	21,111.5	322
48	male	60	21,103.0	284
49	female	78	20,932.5	373
49	male	54	20,899.5	258
50	female	64	20,872.5	307
50	male	47	20,698.0	227
51	female	73	20,785.5	351
51	male	41	20,434.5	201
52	female	73	20,593.5	354
52	male	41	20,184.0	203
53	female	63	20,383.0	309
53	male	39	19,930.5	196
54	female	59	20,319.0	290
54	male	35	19,722.5	177
55	female	59	20,235.5	292
55	male	43	19,500.0	221
56	female	45	20,070.0	224
56	male	47	19,236.0	244
57	female	51	19,830.0	257
57	male	42	19,028.0	221
58	female	68	19,440.5	350
58	male	34	18,676.5	182
59	female	52	19,053.5	273
59	male	52	18,291.0	284
60	female	53	18,631.0	284
60	male	39	17,973.5	217
61	female	44	18,128.0	243
61	male	26	17,556.5	148
62	female	45	17,598.5	256
62	male	28	17,052.0	164
63	female	0	17,059.0	0
63	male	0	16,621.5	0
64	female	0	16,566.0	0
64	male	0	16,051.0	0
65	female	0	16,019.5	0
65	male	1	15,442.5	6
66	female	0	15,407.5	0
66	male	1	14,743.0	7
67	female	0	14,851.5	0
67	male	1	14,102.5	7
68	female	0	14,137.0	0
68	male	1	13,371.5	7
69	female	0	13,453.5	0
69	male	2	12,649.5	16
70	female	0	12,771.5	0
70	male	0	11,956.5	0
71	female	1	12,120.5	8
71	male	0	11,264.5	0
72	female	0	11,437.0	0
72	male	0	10,583.0	0
73	female	0	10,810.5	0
73	male	0	9958.5	0
74	female	0	10,254.5	0
74	male	0	9308.5	0
75	female	0	9743.5	0
75	male	0	8627.5	0
76	female	1	9217.5	11
76	male	0	8005.0	0
77	female	0	8691.0	0
77	male	0	7418.5	0
78	female	0	8235.0	0
78	male	0	6882.0	0
79	female	2	7843.0	26
79	male	0	6406.0	0
80	female	0	7406.0	0
80	male	0	5922.5	0
81	female	0	6997.0	0
81	male	0	5507.5	0
82	female	0	6583.0	0
82	male	0	5062.0	0
83	female	0	6221.0	0
83	male	0	4610.5	0
84	female	0	5838.5	0
84	male	1	4185.0	24
85	female	0	5375.0	0
85	male	0	3746.0	0
86	female	0	4975.0	0
86	male	0	3296.0	0
87	female	0	4620.5	0
87	male	0	2872.5	0
88	female	0	4146.0	0
88	male	0	2447.0	0
89	female	0	3639.0	0
89	male	0	2065.5	0
90	female	0	3121.0	0
90	male	0	1660.5	0
91	female	0	2671.0	0
91	male	0	1307.0	0
92	female	0	2219.5	0
92	male	0	1012.5	0
93	female	0	1806.0	0
93	male	0	766.0	0
94	female	0	1387.0	0
94	male	0	528.5	0
95	female	0	996.0	0
95	male	0	376.5	0
96	female	0	695.0	0
96	male	0	245.0	0
97	female	0	470.5	0
97	male	0	152.5	0
98	female	0	318.0	0
98	male	0	97.0	0
99	female	0	214.0	0
99	male	0	54.0	0
100	female	0	141.0	0
100	male	0	34.0	0
101	female	0	82.0	0
101	male	0	19.0	0
102	female	0	51.5	0
102	male	0	11.5	0
103	female	0	34.5	0
103	male	0	7.0	0
104	female	0	21.0	0
104	male	0	4.5	0
105	female	0	10.0	0
105	male	0	0.0	NaN
106	female	0	9.0	0
106	male	0	0.0	NaN
107	female	0	5.5	0
107	male	0	0.0	NaN
108	female	0	2.0	0
108	male	0	0.0	NaN
109	female	0	1.0	0
109	male	0	0.0	NaN

^1^ Incidence per 100 000 person-years.
